# Influence of Peri-duodenal Non-constrictive Cuff on the Body Weight of Rats

**DOI:** 10.1007/s11695-014-1519-0

**Published:** 2014-12-06

**Authors:** Xiao Lu, Samer G. Mattar, Ghassan S. Kassab

**Affiliations:** 1 Department of Biomedical Engineering, Indiana University Purdue University Indianapolis, Indianapolis, IN 46202 USA; 2 Department of Surgery, Indiana University Purdue University Indianapolis, Indianapolis, IN 46202 USA; 3 Cellular and Integrative Physiology, Indiana University Purdue University Indianapolis, Indianapolis, IN 46202 USA; 4 Indiana Center for Vascular Biology and Medicine, Indiana University Purdue University Indianapolis, Indianapolis, IN 46202 USA; 5 Department of Surgery, Oregon Health & Science University, Portland, OR 97239 USA; 6 California Medical Innovations Institute, 11107 Roselle, San Diego, CA 92121 USA

**Keywords:** Obesity, Cuff, Duodenum, Weight loss, Mechanotransduction

## Abstract

**Background:**

Weight loss has been found to improve or resolve cardiovascular comorbidities. There is a significant need for reversible device approaches to weight loss.

**Methods:**

Non-constrictive cuff (NCC) is made of implantable silicone rubber with an internal diameter greater than the duodenum. Ten or 11 NCC were individually mounted along the duodenum from the pyloric sphincter toward the distal duodenum to cover ~22 mm in the length. Twelve Wistar rats were implanted with NCC, and six served as sham, and both groups were observed over 4 months. Six rats with implant had their NCC removed and were observed for additional 4 weeks.

**Results:**

The food intake decreased from 40.1 to 28.1 g/day after 4 months of NCC implant. The body weight gain decreased from 1.76 to 0.46 g/day after 4 months of NCC implant. The fasting glucose decreased from 87.7 to 75.3 mg/dl at terminal day. The duodenal muscle layer covered by the NCC increased from 0.133 to 0.334 mm. After 4 weeks of NCC removal, the food intake, body weight gain, and fasting glucose recovered to 36.2, 2.51 g/day, and 83.9 mg/dl. The duodenal muscle layer covered by the NCC decreased to 0.217 mm.

**Conclusion:**

The NCC implant placed on the proximal duodenum is safe in rats for a 4-month period. The efficacy of the NCC implant is significant for decrease in food intake, body weight gain, and fasting glucose in a normal rat model. The removal of NCC implant confirmed a cause-effect relation with food intake and hence body weight.

## Introduction

Obesity increases the risk of diabetes, stroke, hypertension, heart disease, kidney disease, liver disease, gallbladder disease, degenerative diseases, and depression, among other disorders [1, 2]. Weight loss has been found to improve or resolve these comorbidities [3–10]. Surgical intervention provides effective treatment for severe obesity [6–11]. Bariatric surgery (e.g., Roux-en-Y gastric bypass, RNY; adjustable gastric banding, AGB; vertical sleeve gastrectomy, VSG; biliopancreatic diversion; and duodenal-jejunal exclusion) based on gastrointestinal restriction or malabsorption provides sustainable, profound weight loss [5–17]. The emerging evidence in bariatric surgery that surgical manipulations of the gastrointestinal anatomy can bring about weight-independent improvements in glycemic control have led to interest in the gastrointestinal tract as a target for the development of new treatments for obesity and diabetes [18–28], albeit the mechanisms of weight loss with many of these procedures are not fully understood. Animal studies suggest that exclusion of chyme from the mucosa and mucosal/submucosal secretions in the proximal small intestine directly and positively affects glucose homoeostasis [8, 15–20]. This finding has prompted the development of devices aiming to mimic the foregut exclusion of bypass procedures [27, 28].

Here, we developed a reversible intervention to achieve weight loss. We previously observed acute reduction of duodenal motility by peri-duodenal non-constrictive cuff [29]. In this study, we implanted an external peri-duodenal non-constrictive cuff (NCC) around the proximal duodenum adjacent to the pyloric sphincter. The NCC did not result in physical stenosis since the internal diameter of the NCC was greater than the duodenal major diameter at physiological distension. The application of the NCC acutely reduces duodenal contractility during elevation of intraluminal pressure [29]. The safety, efficacy, and reversibility of the implantation of NCC were evaluated over a 4-month period.

## Materials and Methods

Eighteen Wistar rats weighing 495 ± 27 g (472–523 g) were obtained from Charles River. The animals had ad libitum access to water and food and were randomly distributed in sham group (*n* = 6), cuff implantation group (*n* = 6), and cuff removal group after the same period of the implantation group (*n* = 6). A room temperature of 20 to 22 °C and humidity of 30 to 70 % were maintained. The animals were carefully checked for preexisting disease and acclimated for a week before undergoing the surgical procedure. All animal experiments were performed in accordance with national and local ethical guidelines, including the principles of laboratory animal care, the Guide for the Care and Use of Laboratory Animals and the National Society for Medical Research, and an approved Indiana University School of Medicine IACUC protocol regarding the use of animals in research.

**Fig. 1:**
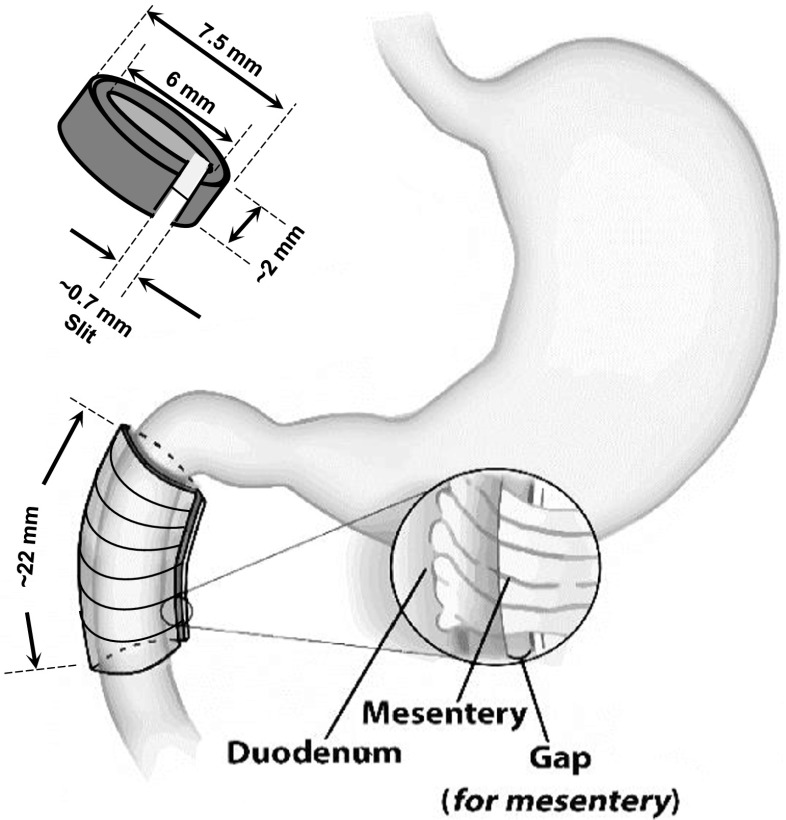
Schematic of NCC implanted on the proximal duodenum. *Left top panel* shows a NCC with dimensional parameters. *Right panel* shows the position and region of NCC implant where the NCC was implanted around the proximal duodenum adjacent to the pyloric sphincter

An NCC was made of implantable silicone rubber, and the wall of NCC was impermeable. The internal and external diameters of NCC were 6 and 7.5 mm, respectively, with axial length of ~2 mm (Fig. 1). The NCC can be opened into a sector for implantation with axial slit of 0.7 mm. The axial gap through the NCC allows the mesentery to pass through the NCC without injury or damage to preserve the blood vessels and neural fibers of the intestine (Fig. 1). Ten or 11 NCCs were implanted in tandem along the small intestine to cover a desired length of the small intestine (Fig. 1).

Surgical anesthesia was induced with and maintained with isoflurane 1.3–1.6 %. Ventilation was provided with a respirator (Harvard Instrument, Inc), and a heating pad was used to maintain body temperature. The adequacy of anesthesia was confirmed by stability of respiration rate and no limb withdrawal reflex. The abdominal skin and muscle layers were incised at a central line. The stomach and proximal duodenum were exposed to provide access for the implant. In the implantation group, ten or 11 NCC were opened by forceps and slipped into the space between the duodenum and right hepatic lobe. The NCC were individually mounted along the duodenum which covered ~22 mm of duodenum length from the pyloric sphincter toward the distal duodenum. In the removal group, all NCC were removed surgically after 4 months, and the animals were survived for additional 4 weeks. The abdominal muscular layer was sutured using absorbable suture, and the skin was closed by silk suture. In the sham group, six pieces of silicone rubber ~4 × 2 × 1.5 mm (L × W × H) were placed on surface of duodenum. The rats were recovered and had free access to food and water.

The activity and behavior of rats were evaluated daily. The food intake and body weight were measured weekly. The incremental food intake or body weight per day was calculated weekly and divided by seven. The fasting glucose in the implant group was measured at 16 weeks and removal group, at 20 weeks.

At termination day, the animals were anesthetized with isoflurane, and a blood sample was collected from the jugular vein. The abdominal skin and muscle layers were reopened. The animal was sacrificed with an overdose of pentobabital. The stomach and small intestine were excised and immediately stored in 4 °C physiological saline solution (PSS) (in mmol/l, 142 NaCl; 4.7 KCl; 2.7 Sodium HEPES, three HEPES acid, 1.17 MgSO_4_, 2.79 CaCl_2_, and 5.5 glucose). The covered duodenum was separated carefully from the NCCs. A ~6-mm segment of the covered duodenum was fixed in formalin (10 %) for histological analysis. Similarly, a ~6-mm segment of uncovered duodenum, proximal and distal jejunum, and ileum were fixed in formalin for histological analysis. Finally, a ~8-mm segment of lower stomach was fixed in formalin for histological analysis.

The fixed samples were embedded in paraffin, and 5-μm sections were placed on slides. Hematoxylin and eosin staining was performed for morphometric measurement. For each segment, 4~5 slides were analyzed. A minimum of ten well-oriented wall thicknesses of mucosa and muscle layers were measured.

### Statistical Analysis

The data were presented as mean ± SD, and significant differences between two groups was determined by Student’s *t* test (two-tailed distribution, two-sample unequal variance). Significant differences between the dose-response groups were determined by the use of Bonferroni post hoc test following analysis of variance (ANOVA) between groups. A probability of *p* < 0.05 was considered to be indicative of a statistically significant difference.

## Results

**Fig. 2:**
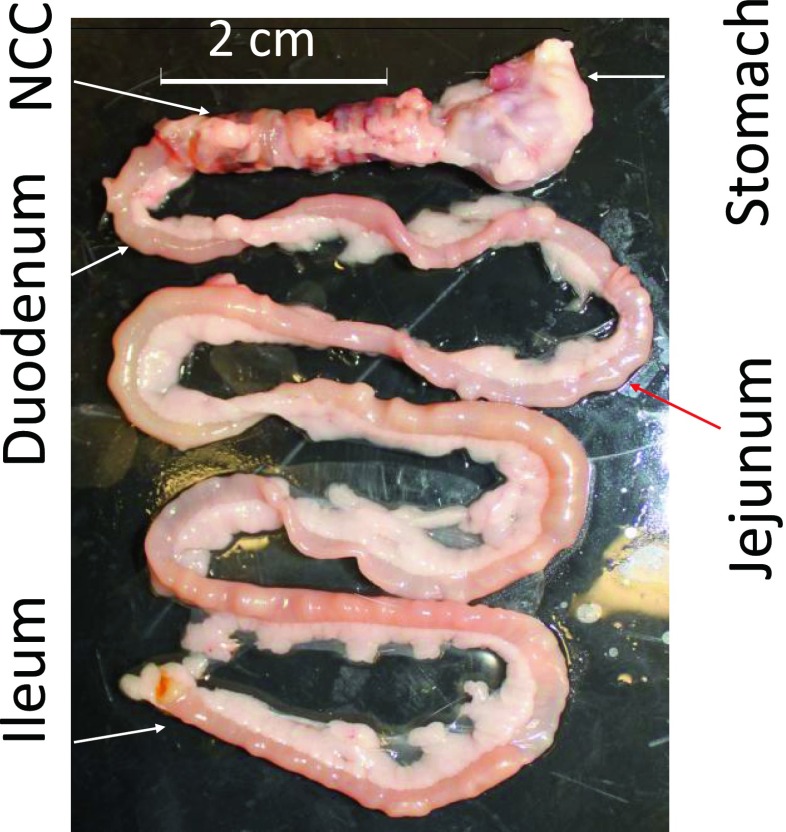
A typical in vitro image of the stomach and small intestine with NCC implant. The stomach and intestine are clear. The implant was embedded in connective tissue. No significant scarring tissue was found

**Fig. 3:**
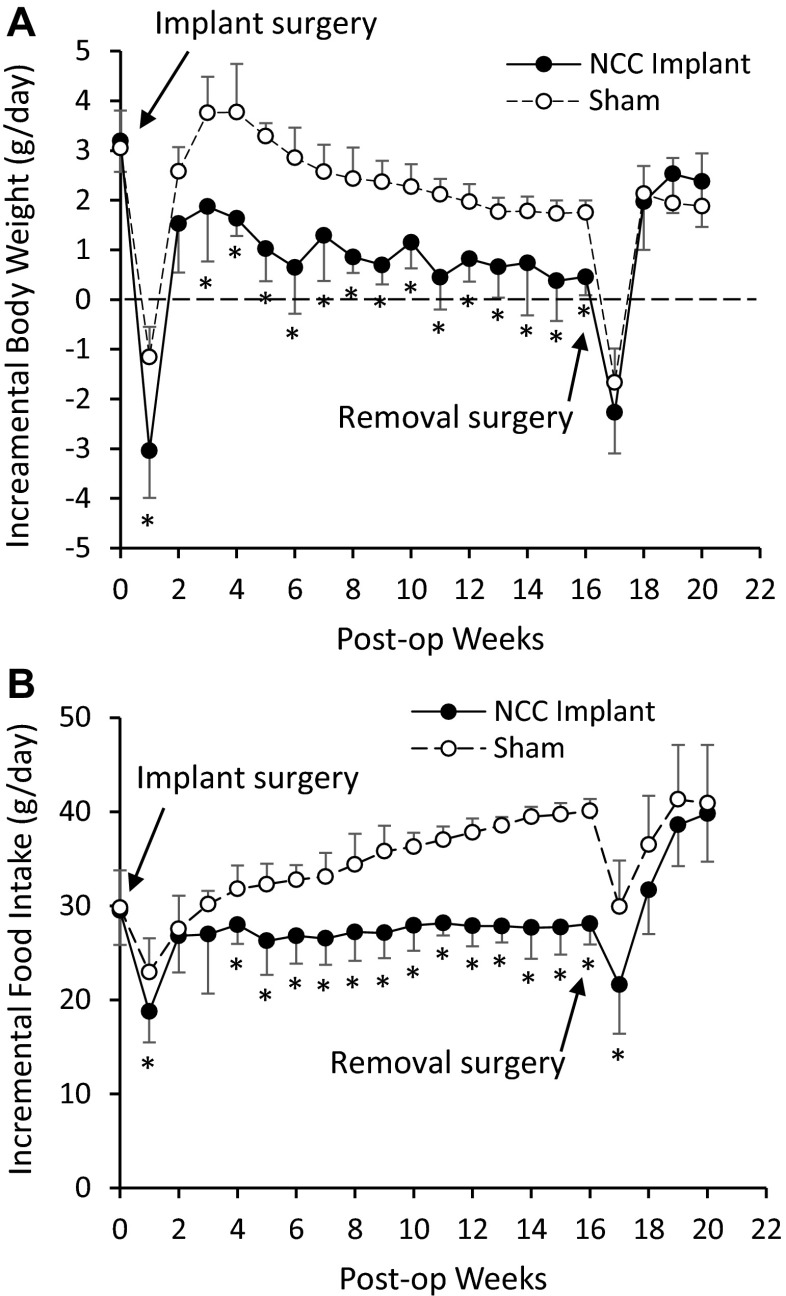
Growth observations in the study period. **a** Body weight gain was also retarded by the NCC implant. The NCC removal significantly recovered the gain. **b** Food intake was significantly reduced by the NCC implant. The food intake was reversed by NCC removal. #: *p* < 0.05 ANOVA followed by post hoc Bonferroni. (*Asterisk*) *p* < 0.05 in comperison with sham

**Fig. 4:**
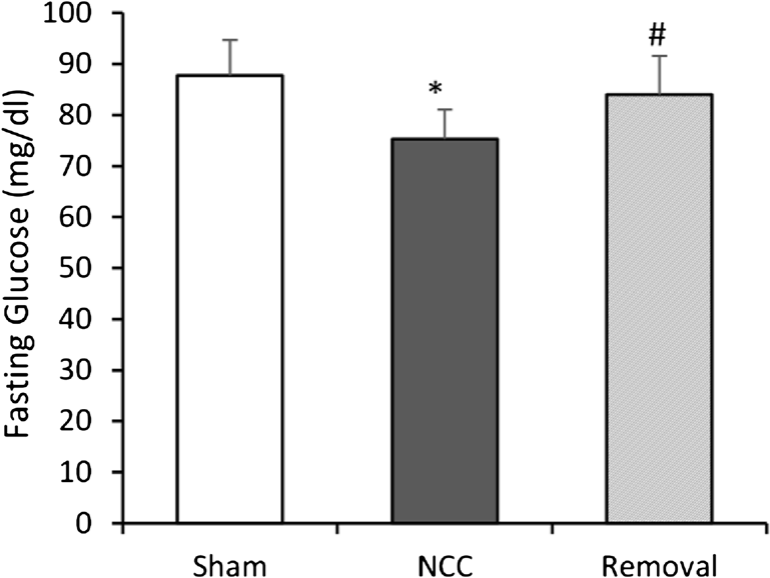
Fasting glucose in the implant. The NCC implant significantly suppressed fasting glucose after 4 months. The fasting glucose was reversed by NCC removal. (*Asterisk*) *p* < 0.05 Student’s *t* test in comparison with sham

**Fig. 5:**
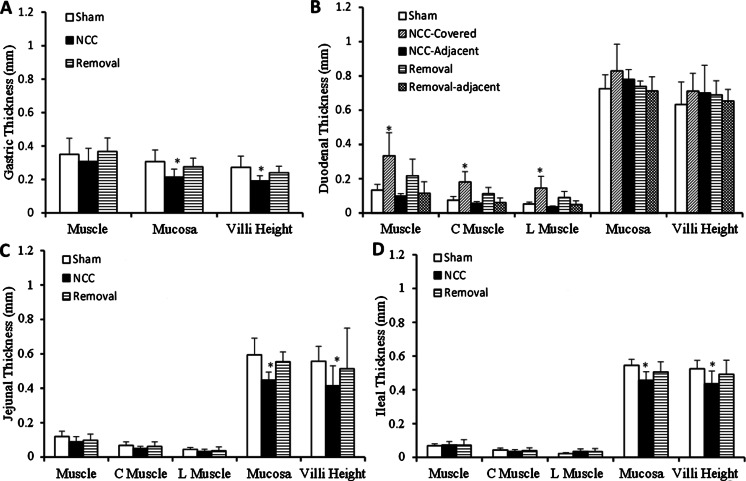
Morphometric measurements of stomach and small intestine. **a** Stomach. Both mucosa and villi height decreased in the stomach and were reversed by the removal of NCC. **b** Duodenum. Muscle thickening is significant. Both circumferential and longitudinal muscle layers were proportionally thickened. The removal of the implant reversed muscle thickening. **c** Jejunum. **d** Ileum. Both mucosa and villi height decreased in the jejunum and ileum and were reversed by the removal of NCC. (*Asterisk*) *p* < 0.05 Student’s *t* test in comparison with sham

**Fig. 6:**
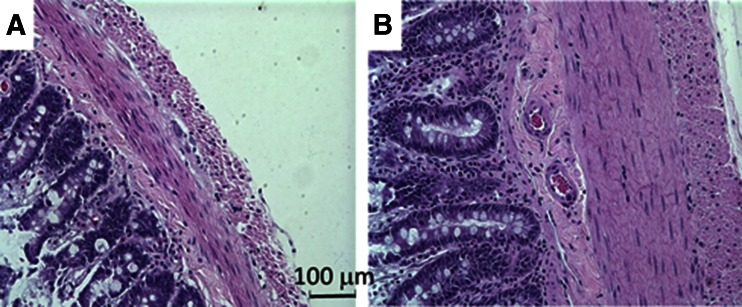
Histologic slides of the duodenal segment in the NCC. **a** Sham. **b** The NCC covered. ×20 objective. The muscle layers covered by the NCC were approximately equal to those of sham. The average distend between the nuclei of smooth muscle cells increased significantly

After ~4 days post-op, the activity of rats was completely restored to the level before implantation. No distress was found in any implanted rats. Water consumption did not change (40–50 ml/day) after NCC implantation. The behavior of rats did not change after NCC implantation. There were not any signs of pain or distress in any rats. At termination study, no inflammatory response was found in the gastrointestinal system. A typical gastrointestinal image with the NCC implant was presented in Fig. 2. The NCC implant significantly changed food intake and body weight of rats as compared to sham. In the sham group, the daily increase in food intake and body weight reflected normal growth of rats (Fig. 3). In the NCC implant group, the daily food intake significantly decreased in comparison with the sham group (Fig. 3a). The body weight gain significantly retarded as compared to normal growth in the sham group (Fig. 3b). We found that fasting glucose in plasma was lower in the NCC implant group as compared to the sham (Fig. 4). A decrease in mucosal layer and villi height of the gastric wall was observed, and the thickness of gastric muscle layers did not change significantly (Fig. 5a). The proximal duodenal muscle layer that was covered by the NCC significantly thickened both in the circumferential and the longitudinal muscle layers (Fig. 5b). Furthermore, the thickening of the duodenal segment covered by the NCC was mainly a result of smooth muscle hypertrophy (Fig. 6). The distal duodenal muscle layer that was not covered by the NCC did not change significantly, albeit it tended to decrease (Fig. 5b). The mucosa thickness and villi height did not change in either proximal (NCC-covered) or distal (no NCC) duodenum (Fig. 5b). The jejunal ileal muscle layers did not change in NCC implant (Fig. 5c, d). The jejunal and ileal mucosa thickness and villi height significantly decreased in NCC implant in comparison with sham (Fig. 5c, d).

To examine the cause-and-effect relation, we removed the NCC after 16 weeks of implant. Although fibrotic tissue covered the NCC, the duodenal serosa covered by the NCC did not stick to the internal or external surface of the NCC. The NCC was easily removed by a ~2-mm incision along the fibrotic tissue. After 4 weeks from the removal, the daily incremental food intake and body weight increased to similar levels of the sham group (Fig. 3). The fasting glucose was no longer statistically lower than the sham group (Fig. 4). The morphometric data also tended to recover to sham levels (Figs. 5 and 6). As expected, the body weight (593 g) in the removal group was significantly lower than that in the sham group (669 g) since the weight gain in the removal group was 16 weeks behind the sham group.

## Discussion

The safety and efficacy of novel NCC implants on proximal duodenum were evaluated. The NCC implants did not result in any notable safety risk for the duration of 16 weeks. The food intake, body weight, and glucose of the rats were suppressed in the NCC group. The thickness of the duodenal muscle layer covered by NCC doubled while the mucosal thickness of the jejunum and ileum decreased significantly. The removal of NCC reversed food intake, body weight, as well as the morphometric parameters of the small intestine. The recovery of the former supports a cause-and-effect relation for the device while restoration of the latter suggests reversible non-pathological remodeling.

Body weight is the result of complex physiologic mechanisms that control food intake and energy expenditure in which neural and hormonal signals are involved [15]. Control of body weight is a significant topic in the design of effective treatments for obesity. Bariatric surgery provides decrease in body weight and has become a common treatment for obesity. It is currently recognized as an effective treatment for prevention, control, or reversion of the comorbidities including metabolic disease [18–25]. While multiple surgical approaches, including RNY, VSG, and laparoscopic AGB, have shown efficacy in achieving weight loss and improving/eliminating obesity-related conditions, the bariatric procedure penetration rate remains quite low at approximately 1 % of the obese population. A significant reason for low penetration rate is patient reluctance because of the many shortcomings of the available procedures [30, 31]. The most notable shortcomings include permanence or irreversibility, staple line leak complications, and nutritional deficiencies [30, 32, 33], in the case of purely surgical approaches. In the case of AGB, the complications include frequent adjustment requirements, esophageal distention (including achalasia), and insufficient weight loss, leading to up to 50 % band removal as recently reported [32, 33]. The proposed NCC provides a number of advantages including efficacy in the limitation of food intake and body weight, reversibility, elimination of the staple-related complications, no esophageal distensions, and no indications of gastrointestinal rupture.

Pyloric adjustable banding, an AGB implanted at the pyloric sphincter, has been employed to regulate gastric emptying rate [34]. Food intake reduction and weight loss were observed after inflation of the pyloric band. The incidence of gastric dilation, however, was high [34] because the pyloric adjustable banding creates a significant physical obstruction which is different from the NCC employed in this study. The diameter of NCC is 6 mm which is ~20 % larger than the duodenal diameter (~5 mm), and it was placed in the proximal duodenum from the pyloric sphincter toward the middle duodenum. The mechanism NCC efficacy is unclear and requires further investigation. Although NCC is not physically obstructive, it may acutely attenuate duodenal contractility in response to pressure stimulus [29]. The reduction of motility may extend, however, beyond the covered portion [35, 36]. Furthermore, the motility reduction at the covered region likely transmits feedback to the entire GI system [37–42]. It is well-known that a reduction in motility may produce two major results: (1) increase of the sustained time of chyme in the stomach or a delay of the gastric emptying (satiety persistence) and (2) mixing reduction of nutrient chyme in the duodenum for delivery of premature nutrients to the jejunum (malabsorption).

The NCC-induced duodenal remodeling thickens the muscle layer covered by NCC and did not affect the duodenal mucosa (Fig. 5a). The regional thickening of the duodenal muscle covered by NCC is interesting since the muscle layer of contiguous distal duodenum is not thickened. The effect of NCC on muscle thickness is limited to the region of NCC where the contraction is attenuated [29]. The mechanism of muscle thickening is unclear, and it is likely compensatory to the reduced contractility since the NCC attenuates duodenal motility. We also observed significant increase in the duodenal lumen at NCC implant sites. This may be evidence of duodenal motility attenuation since chyme transportation merely depends on passive pressure difference between the stomach and distal NCC. We also observed that the NCC implant decreased jejunal and ileal mucosal thickness (Fig. 5b, c) which suggests that the effect of NCC extends along the entire small intestine. This is in agreement with bariatric experience where the effect is not regional but global. The NCC implant also significantly affected gastric morphometric parameters (Fig. 6) likely due to the increased resistance of chyme transportation. Therefore, the NCC implant induces some gastrointestinal remodeling likely as adaption of gastric motility similar to bariatric surgery but with the advantage of reversibility.

The removal of the NCC was rather simple with no complications. We found restoration of food intake, body weight gain, intestinal muscle, and mucosal layers post removal. Since the NCC implant does not change the gastrointestinal anatomy, reversibility was expected after NCC removal.

Although we demonstrated that gastrointestinal contraction is acutely attenuated by NCC, it remains whether NCC implant chronically attenuates gastrointestinal motility, e.g., chyme dynamical transportation, bolus transient time, etc. The understanding of efficacy requires future study of gut pathway activation, particularly the peptide hormone secretions, non-peptide signals transported across the mucosa, enteric neuronal circuits and communication, and autonomic and central nervous system interactions. The efficacy of NCC implant should also be validated in animal models of metabolic syndrome, e.g., obesity and type II diabetes.

In summary, the NCC implant on proximal duodenum is safe in rats for 4 months. The efficacy of the NCC implant was demonstrated by the decrease in food intake, body weight gain, and fasting glucose. The gastrointestinal remodeling and adaptation was substantially restored after NCC removal. The reversibility of the NCC implant also restored food intake, body weight gain, and fasting glucose, which confirmed causation of the implant. Understanding the mechanism of NCC implant may lead to new strategies and approaches for the treatment of obesity and diabetes.
